# The Occurrence of Potential Harmful Cyanobacteria and Cyanotoxins in the Obrzyca River (Poland), a Source of Drinking Water

**DOI:** 10.3390/toxins12050284

**Published:** 2020-04-28

**Authors:** Wanda Czyżewska, Marlena Piontek, Katarzyna Łuszczyńska

**Affiliations:** 1 Water and Sewage Laboratory, Water and Wastewater Treatment Plant in Zielona Góra, Zjednoczenia 110 A, 65-120 Zielona Góra, Poland; wanda.czyzewska@zwik.zgora.pl; 2 Institute of Environmental Engineering, University of Zielona Góra, Licealna 9, 65-417 Zielona Góra, Poland; k.luszczynska@iis.uz.zgora.pl

**Keywords:** cyanobacterial bloom, cyanotoxins, biotoxicological test *Dugesia tigrina*, source of drinking water

## Abstract

Harmful cyanobacteria and their cyanotoxins may contaminate drinking water resources and their effective control remains challenging. The present study reports on cyanobacterial blooms and associated cyanotoxins in the Obrzyca River, a source of drinking water in Poland. The river was examined from July to October 2019 and concentrations of microcystins, anatoxin-a, and cylindrospermopsin were monitored. The toxicity of water samples was also tested using an ecotoxicological assay. All studied cyanotoxins were detected with microcystins revealing the highest levels. Maximal microcystin concentrations (3.97 μg/L) were determined in September at Uście point, exceeding the provisional guideline. Extracts from Uście point, where the dominant species were *Dolichospermum flos-aquae* (August), *Microcystis aeruginosa* (September), and *Planktothrix agardhii* (October), were toxic for *Dugesia tigrina* Girard. Microcystin concentrations (MC-LR and MC-RR) were positively correlated with cyanobacteria biovolume. Analysis of the chemical indicators of water quality has shown relationships between them and microcystins as well as cyanobacteria abundance.

## 1. Introduction

Global warming and water pollution by nutrients that cause dangerous cyanobacterial blooms are an increasingly common problem, especially in drinking water sources [1]. The presence of toxic cyanobacteria and microcystins in drinking water sources is a widespread phenomenon [2]. Cyanotoxin problems in drinking water treatment plants (DWTPs) are observed in many countries around the world, for example the United States (North America), Brazil (South America), Vietnam, Saudi Arabia (Asia), Egypt (Africa), and Australia [2,3,4,5,6]. Additionally, in Europe, problems with cyanobacteria metabolites in drinking water are still current [7,8,9].

Apart from making water treatment more difficult (clogging of filters), cyanobacterial blooms may deteriorate the quality of water [10]. Cyanobacteria can produce a large number of secondary metabolites. Odorous metabolites affect the smell and flavor of drinking water and of aquatic animals, where bioactive metabolites cause a range of lethal and sub-lethal effects in plants, invertebrates, and vertebrates, including humans. Due to structural similarity, the 157 known bioactive classes produced by cyanobacteria have been recently condensed to 55 classes [11].

Cases of poisoning, lethal even for animals and people, by cyanobacterial toxins, have been observed in various regions of the world [12,13,14,15,16,17]. Chronic exposure to low doses of selected cyanotoxins can be associated with organ damage (e.g., liver, kidney) [18,19] or neurotoxicity [13].

Conventional drinking water treatment involving filtration, flocculation, and disinfection reduces, but does not always eliminate cyanobacteria and their metabolites including cyanotoxins. The disruption of cells during either physical (e.g., filtration, sedimentation) or chemical treatment (e.g., disinfection) and the subsequent release of toxins warrants the significant concern of water treatment plants (WTP) operators and water supply managers [10,20,21].

Nevertheless, a monitoring of water bodies and supply systems for cyanobacteria and cyanotoxins is not yet common practice in most countries in the world. Therefore, illness directly caused by cyanobacteria toxins [16] and the effects of chronic low-dose exposures are challenging to assess and may be underestimated [19]. There are a number of critical control points in the potable water supply system where testing for cyanotoxin and intact cyanobacterial cells should be carried out if significant cyanobacterial densities occur in the source water. These may include the water storage reservoir or river [21].

A number of countries have developed regulations and guidelines for cyanotoxins and cyanobacteria in drinking water [22]. The World Health Organization (WHO) has recommended a provisional limit value of 1 µg/L MC-LR in drinking water [23]. Countries that have adopted the WHO provisional guideline for MC-LR for drinking water directly include Czech Republic, France, Japan, Korea, New Zealand, Brazil, and Spain. Unfortunately, determination of MC-LR in surface water, being a source of drinking water, was not recommended in Poland until 2007 [24]. Based on toxicology, epidemiology, and occurrence studies, the United States Environmental Protection Agency (EPA) Office of Ground Water and Drinking Water recommended the cyanotoxins MC-LR, MC-YR, MC-RR, MC-LA, anatoxin-a (ANA), and cylindrospermopsin (CYN) to be placed on the Unregulated Contaminant Monitoring Rule, which is used as a program to collect data for contaminants suspected to be present in drinking water. This monitoring supplies information on the nature and size of populations exposed to cyanotoxins through tap water [25]. Although cyanobacterial blooms typically occur in lakes and reservoirs, incidents of cyanobacterial blooms were also observed in rivers as well, e.g., Nile River (Egypt) [26], Narew, Obrzyca River (Poland) [9,27].

**Figure 1:**
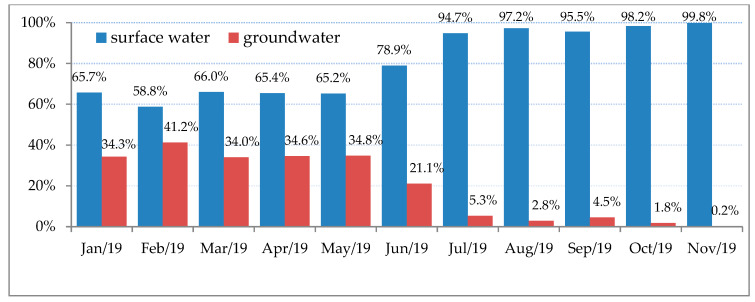
Percentage share of particular water sources in the consumption water supply for the inhabitants of Zielona Gora in 2019.

The present study aimed to describe the concerns regarding the potential problem of cyanobacterial blooms in raw water treated in the DWTP in the Zielona Góra (Lubuskie Province, central-western Poland), where one of source of drinking water is surface water from the Obrzyca River. In previous years in the DWTP, ground and surface water were mixed, which was one of the solutions to eliminate hepatotoxins [9]. As a result of drought and high temperature in the summer, the amount of groundwater significantly decreased in 2019. Therefore, the share of treated surface water increased in the second half of the year, as presented in Figure 1.

Following the latest trend in scientific works aimed at explaining a threat of cyanotoxins in drinking water to human health and environment [2,4,6,7,8,9,16,18,19,21] and based on the earlier conducted studies [9,17] further research was undertaken in the Obrzyca River in the hydrological season of 2019. The studies included extended scope of cyanotoxin analyses (besides microcystins, anatoxin-a and cylindrospermopsin were investigated). Based on the results of 2017 [9] concerning the places and months of occurrence of cyanobacterial blooms in the tested catchment areas, the two sampling points were selected, which were the most exposed to cyanobacterial blooms—Uście and Wojnowo, and one nearer the DWTP, Sadowo. In order to evaluate the toxicity of tested samples, a sensitive bioassay using the planarian *Dugesia tigrina* was conducted [17,28].

## 2. Results

### 2.1. Cyanobacterial Blooms

The amount of cyanobacteria varied between the three sampling points. The maximum biovolume of cyanobacteria exceeded 88.5 mm^3^/L and occurred at point Uście in September 2019 (Table 1). At the same point, cyanobacterial blooms (chlorophyll a above 20 µg/L) were present throughout the entire research period. The dominant species at Uście point was *Dolichospermum flos-aquae* in July (96% of cyanobacteria biovolume). Co-dominance of *Microcystis aeruginosa* and *D. flos-aquae* occurred in August. *M. aeruginosa* was dominant (89%) in September and *Planktothrix agardhii* in October. Moreover, in the sample from October, significant abundance (27%) of *M. aeruginosa* was observed. A chlorophyll a concentration above 20 µg/L was also found at Wojnowo point, but only in two months: August and September, where dominant species *D. flos-aquae* and *Oscillatoria angusta* occurred. Furthermore, in the Wojnowo sample, two cyanobacterial species were observed with percentages above 10% of total cyanobacteria biovolume, *Microcystis wesenbergii* in October (31%) and *Woronichinia naegeliana* in July (25%), August (18%), and September (13%). The lowest concentrations of chlorophyll a and cyanobacteria biovolume were at point Sadowo. Dominant species at this point were: *Pseudanabaena limnetica* in August and September and *M. aeruginosa* in October (85%). Other species observed in the analyzed samples, however with low percentage (<5%) of cyanobacteria community, were: *Dolichospermum planctonicum*, *Aphanizomenon flos-aquae*, *Microcystis viridis*, and *Cuspidothrix issatschenkoi.*

Analysis of variance showed highly significant differences between sampling points and cyanobacteria biovolume (F_calc_ 75.1; F_crit_ 8.02; *p* > 0.01).

### 2.2. Cyanotoxins

The maximal value of intracellular cyanotoxins (1.66 μg/L dmMC-RR) occurred in October at the Uście point (Table 2). A microcystin value of >1 μg/L was detected in September at the Uście point (1.33 μg/L MC-LR, 1.56 μg/L dmMC-RR). Concentrations of cyanotoxins above 1 μg/L were not detected at the rest of the sampling points (Wojnowo and Sadowo). The maximal concentration of intracellular anatoxin-a occurred at the Wojnowo point in September and equaled 0.56 μg/L. Nodularin and MC-LA were not detected in the tested samples. The concentration of intracellular cylindrospermopsin was below 0.05 μg/L in the analyzed samples.

### 2.3. Bioassay Test

Samples collected from July to October at the Uście point were toxic for *D. tigrina*. The rest of the tested samples were not toxic for the planarian (Table 3, Supplementary Materials: Tables S2–S7).

Among toxic samples 240 h LC 50 was in the range of 20.9–41.7% out of the analyzed extracts’ concentration. The most toxic sample was in September, where LC 50 amounted to almost 21%.

### 2.4. Physical and Chemical Water Quality Indicators

In this study, 10 water quality indicators were analyzed in terms of their impact on the biovolume of cyanobacteria and their toxin synthesis. The statistical analysis is presented in Table 4. A correlation was observed between cyanobacteria biovolume and pH, turbidity, total nitrogen, and total suspension. Furthermore, a strong correlation was observed between microcystins and the following water quality indicators: total phosphorus, total nitrogen, turbidity, and total suspension. A correlation was observed between pH and MC-LR and MC-RR.

**Figure 2:**
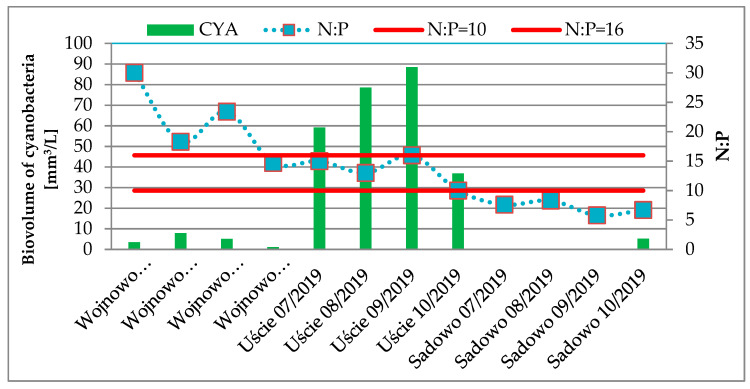
Biovolume of cyanobacteria vs. N:P ratio in the Obrzyca River at sampling points.

No correlation was observed between cyanobacteria biovolume and N:P ratio, but our results revealed that cyanobacterial blooms occurred when the N:P ratio was between 10 and 16 (Figure 2). Cyanobacterial blooms occurred at the Uście point in the whole sampling period where N:P ratio ranged from 10 to 16. The N:P ratio equaled 15 in October at Wojnowo point, but there were no cyanobacterial blooms, this is explained in the discussion. 

## 3. Discussion

Massive cyanobacterial blooms are the result of eutrophication of the water environment. At the same time, they create problems not only during water treatment by clogging filters, disinfection by-products, taste, odor, etc. [29], but toxic cyanobacterial blooms also have a negative impact on human health. Therefore, monitoring cyanobacterial biomass in surface waters and especially drinking water sources is crucial in quality control systems [9]. Studies have shown that the most exposed location to cyanobacterial blooms in the Obrzyca River is the Uście point.

During the whole study period, the biovolume of cyanobacteria at the Uście point, exceeded 10 mm^3^/L. The threshold for Alert Level 2 (10 mm^3^/L biovolume or 50 μg/L chlorophyll a with the presence of toxins confirmed by chemical or bioassay techniques) is described as established and toxic bloom. An Alert Levels Framework is a monitoring and management action sequence that water treatment plant operators and managers can use to provide a graduated response to the onset and progress of cyanobacterial bloom [21].

Previous studies of the Obrzyca River also indicated Uście as the most exposed point to cyanobacterial blooms [9]. The most probable source for the observed blooms in the Obrzyca River at the Uście point is the upstream located Rudno Lake [30]. Therefore, the next step should be to examine the lake for cyanobacterial blooms and their toxicity.

During massive occurrences of cyanobacteria at the Uście point, the dominant species were *D. flos-aquae* (July, August) and *M. aeruginosa* (September). In October, *P. agardhii* and *M. aeruginosa* were present as co-dominants, as in previous studies [9]. All three species occur frequently in Polish freshwater bodies: dam reservoirs (Zemborzycki, Siemianówka) and lakes (Orle, Białe, Bytyńskie, Lubosińskie, Syczyńskie) [31].

The filamentous cyanobacteria *Planktothrix* spp. are among the most important microcystin producers and can be found in freshwater habitats in temperate regions in the Northern Hemisphere. *P. agardhii* occurs in high abundance in shallow and eutrophic lakes [32].

Bloom-forming and microcystin-producing *P. agardhii* strains were even observed in French lakes in May and November [33]. *P. agardhii* was a dominant species in German lakes in the Scharmützelsee region (East-Brandenburg) [34] and in marine waters off the northwest Portuguese coast [35].

*M. aeruginosa* is a global bloom-forming cyanobacterial species that can produce several types of microcystins [25].

Some species belonging to the genus *Dolichospermum* are confirmed to produce different types of toxins i.e., microcystins, cylindrospermopsin, anatoxins, and saxitoxins [36,37].

During our studies (2019), microcystins were detected and their concentrations were above 1 μg/L for MC-LR and dmMC-RR. The maximal value of total intracellular microcystins (3.97 μg/L) was determined at the Uście point in September where *M. aeruginosa* constituted 89% of cyanobacteria community. Additionally, at the Uście point, a concentration above 1 μg/L dmMC-RR was determined in October. There was in the sample not only *M. aeruginosa* constituting 69% of cyanobacterial biovolume but also *P. agardhii* (27% of cyanobacteria biovolume). In previous studies (2008–2012) [9], intracellular microcystins (expressed as equivalent of MC-LR), ranged from <0.15 to 15.7 μg/L. The highest microcystin concentrations were noticed in September (samples collected from May to September) where dominant species constituted *P. agardhii*, *M. aeruginosa*, or *M. flos-aquae*. In two lakes, Lubosińskie and Bytyńskie, situated in Western Poland, dmMC-RR was detected where a *P. agardhii* bloom occurred [38]. Microcystins dmMC-RR and MC-RR were identified as the major microcystin variants in most samples from *P. agardhii* bloom in the Siemianówka Dam Reservoir [39]. Demethylated microcystin variants, i.e., dmMC-RR, were detected in Lake Chao (China), a highly eutrophicated surface water that is used as a drinking water resource for Hefei City. Sixteen isolated strains of *M. aeruginosa* evidenced that non-toxic, toxic, and highly toxic strains coexisted in the lake [40].

Wojnowo was the sampling point where in September maximal anatoxin-a concentration was determined (0.57 μg/L). During the whole study period, *D. flos-aquae* was present at the sampling point. Anatoxin-a is known to be produced by the freshwater genera: *Aphanizomenon, Dolichospermum, Microcystis, Planktothrix,* and *Raphidiopsis* collected from several geographic areas, i.e., Brazil, Canada, Denmark, the United States [11]. Mass development of *Dolichospermum* sp. was observed mainly in the northern part of Poland. In the lakes of Pomerania Province, the concentration of anatoxin-a did not exceed 6 μg/L [31]. Large and harmful cyanobacterial blooms in two newly built artificial reservoirs (Konstantynów and Kraśnik) were observed with one of the highest concentrations of the toxin reported. In the first year of operations of the smaller Konstantynów Reservoir, the mass development of *D. flos-aquae* and *Planktolyngbya limnetica* (48.7% and 53.6% of the cyanobacterial abundance) occurred in summer. The surface scum developed in summer consisted of *D. flos-aquae* that contained high amounts of anatoxin-a (1412.4 μg/L) and smaller amounts of microcystins (10 μg/L equivalent of MC-LR). Furthermore, neurotoxin (anatoxin-a) was observed (maximal value 43.6 μg/L) in the larger Kraśnik Reservoir [41].

In this study, cylindrospermopsin was detected only at Wojnowo point and at a very low concentration, below 0.05 μg/L. The structure of cylindrospermopsin was elucidated from an Australian freshwater *Raphidiopsis raciborskii* bloom [11]. In Poland the *Raphidiopsis* sp. is not so common as *Microcystis* sp., *Planktothrix* sp., or *Dolichospermum* sp. *R. raciborskii* was documented for the first time in the artificially heated Lake Pątnowskie and later in Lake Licheńskie which is characterized by high water temperatures (reaching 30 °C) and never forming an ice cover. Both lakes are situated near Konin in central-western Poland. The occurrence of this cyanobacterium has also been reported in several thermal natural lakes of Western Wielkopolska [42]. Cylindrospermopsins are produced by the freshwater species belonging to the genera: *Dolichospermum, Aphanizomenon, Cylindrospermopsis, Lyngbya,* and *Oscillatoria* [11]. Cylindrospermopsin produced by *Aphanizomenon gracile* was documented in Polish lakes [13]. The cytotoxin induces inhibition of protein synthesis at the translation step in human cells of liver, kidneys, lungs, heart, stomach, adrenal glands, the vascular system, and the lymphatic system. CYN causes DNA fragmentation and loss of whole chromosomes, e.g., in hepatocytes, lymphocytes [16]. In this study, *A. gracile* was not detected. However, in the samples from Wojnowo *A. flos-aquae* was present, which constituted about 1%–3% cyanobacterial community.

In the present study, a relationship between N:P was found (ranging from 10 to 16) at Uście point. Although at the Wojnowo point in October, the N:P ratio equaled 15, no cyanobacterial blooms were observed, probably because of low water temperature (14.8 °C). Previous studies have shown that cyanobacterial bloom was observed when N:P was in the range of 10–16 and water temperature exceeded 20 °C [9]. Water temperature is consistently one of the most important drivers for cyanobacterial blooms, but it is interrelated with other factors such as seasonal changes in water column stability, light, and nutrient availability [43]. High probability (>50%) of harmful algae blooms may occur when the temperature is in the range of 20–22 °C [44]. TN:TP ratios below 40 favored an increased biomass of *Microcystis* sp. in Peipsi Lake and total cyanobacterial biomass in the Võrtsjãrv Lake (North East Europe) [45]. Studies carried out in 137 lakes located within the state of Iowa (USA) have shown that cyanobacterial blooms were at low TN:TP ratios (<20:1) [46].

The correlation coefficient analysis showed the relationships between water quality indicators and cyanobacteria abundance and their toxins. A correlation was observed between cyanobacteria biovolume and pH, turbidity, total nitrogen, and total suspension. A strong correlation was observed between microcystins and the following water quality indicators: pH (only for MC-RR and MC-LR), total phosphorus, total nitrogen, turbidity, pH, and total suspension. The results obtained in this work concur with our previous studies in the Obrzyca River where a relationship was observed between total nitrogen, total suspension, turbidity, and cyanobacterial abundance.

Beversdorf et al. [47] indicated a correlation between turbidity and microcystin concentration in the Winnebago Lake where *Aphanizomenon* and *Microcystis* blooms were present.

Grabowska and Mazur-Marzec [39] observed a positive correlation between pH and microcystins produced by *P. agardhii* in the Siemianówka Dam Reservoir.

Peretyatko et al. [48] have shown that a pH of 8 is the breakpoint beyond which the probability of cyanobacterial bloom occurrence starts to increase rapidly, reaching 100% at pH > 8.8. This implies that in such conditions, cyanobacterial blooms become virtually inevitable in the ponds studied. On the other hand, the probability of bloom occurrence below the breakpoint is at or close to 0. A pH > 8 was determined in Obrzyca River at the Uście point during the whole analyzed period.

In our study, it was revealed that the presence of microcystins was positively related to total phosphorus and nitrogen concentration in water. Kokociński et al. [49] also determined a relationship between total phosphorus and intracellular microcystins in *P. agardhii* from Lake Lubosińkie.

Lu et al. [50] found that nitrogen and phosphorus metabolism were the top two categories to increase their gene expressions prior to and during a toxic algal bloom. These findings provided evidence that genes associated with nitrogen and phosphorus metabolism played important roles in cyanobacterial bloom formation. It has been assumed that cyanobacterial blooms are a consequence of a synergistic interaction between available nutrients and the microorganisms’ physiological capabilities to use such nutrients under favorable weather conditions. Among nutrients, nitrogen and phosphorus are two of the most important bloom drivers.

There are many available methods to detect and identify cyanobacterial toxins, such as bioassays, biochemical assays, chemical assays, and molecular analyses. At present, there is no single method that is optimal for the detection and identification of all types of cyanobacterial toxins and each method has its applicability. Detection methods are affected by the variety and abundance of cyanotoxins. The choice of method is also inevitably influenced by the availability of analytical equipment and its applicability in a particular environment [37].

The superiority of bioassays over chemical analyses of individual toxins is that they show the complete total toxicity of the sample. Therefore, we compared the effects of natural mixtures of cyanobacterial toxins present in aqueous extracts of cyanobacterial bloom samples on planarian communities (representative of water biocoenosis). The extract from Uście point taken in September was most toxic for *D. tigrina*. In the sample, the co-dominant species were *P. agardhii* and *M. aeruginosa*. Our observations revealed that even a 21% extract concentration was toxic for *D. tigrina,* although the sum of microcystins was below 5 μg/L. Previous studies have shown that planarians such as *D. tigrina* were more sensitive (240 h LC 50, 1.51 mg/L mixture of microcystins MC-LR, MC-YR, MC-RR) than *Daphnia magna* (240 h LC 50, 3.09 mg/L mixture of microcystins MC-LR, MC-YR, MC-RR) [17].

According to DeMott et al., acute toxicity with purified toxins has shown that four species of zooplankton differ markedly in their physiological sensitivity to cyclic peptide hepatotoxins from *M. aeruginosa* (MC-LR). The copepod *Diaptomus birgei* was most sensitive (48 h LC-50 for MC-LR ranging from 0.45 to 1.0 mg/L), *Daphnia pulicaria* was least sensitive (48 h LC 50, 21.4 mg/L), and *Daphnia hyalina* (48 h LC 50, 11.6 mg/L) and *Daphnia pulex* (48 h LC 50, 9.6 mg/L) exhibited intermediate sensitivity [51].

The studies conducted by Pawlik-Skowrońska et al. [52] confirmed a much higher toxicity in cyanobacterial extracts containing mixtures of various cyanotoxins and other cyanobacterial metabolites than of pure MC-LR and anatoxin used in an equivalent concentration. High concentrations (1.66–3.32 mg/L) of pure MC-LR caused an acute toxic effect on *D. pulex*. For *Brachionus calyciflorus,* MC-LR was non-toxic within the range of 0.42–3.32 mg/L. Our experiments revealed a much higher toxicity of the cyanobacterial extracts containing mixtures of various cyanotoxins than the toxicity of pure toxins studied by Pawlik-Skowrońska et al. [52]. The studies focused on the toxicity observed in daphnids and rotifers.

Therefore, our observation confirmed Pawlik-Skowrońska et al.’s [52] suggestion that mass development of cyanobacteria, even those not producing cyanotoxins, e.g., microcystins, should be considered as a potential threat to zooplankton communities.

Although cyanobacteria have not changed their morphological structure for over 3.5 billion years, they are characterized by enormous genetic, physiological, and ecological plasticity caused by climate change or the ongoing eutrophication process. Therefore, it is recommended to constantly monitor the abundance of cyanobacteria and their metabolites in sources of drinking water.

## 4. Conclusions

In summary, the results presented in the manuscript have shown that:

1. The occurrence of cyanobacterial blooms in the Obrzyca River is punctual.
2. The highest intracellular microcystin concentration (3.97 μg/L) was determined in the Obrzyca River at the Uście point, where cyanobacterial bloom was noticed.
3. Cyanobacterial extracts collected from August to October at the Uście point were toxic for *D. tigrina* where intracellular microcystins were present. The most toxic sample for planarians was taken in September (LC 50 was 21% extract concentration). Bioassays with *D. tigrina* are sensitive and applicable for the assessment of the toxicity of cyanobacterial blooms.
4. A correlation between cyanobacterial abundance and pH, turbidity, total nitrogen, and total suspension was found. Water quality indicators, i.e., pH, total phosphorus, total nitrogen, turbidity, and total suspension were strongly correlated with intracellular microcystins MC-RR and MC-LR. The analysis showed that cyanobacterial blooms took place when the N:P ratio was in the range of 10–16.
5. The conducted studies (biological, chemical, and physical) complement each other well and thus are a great tool to analyze the risks of harmful cyanobacteria in drinking water, especially in DWTPs.

## 5. Materials and Methods

### 5.1. Study Area

**Figure 3:**
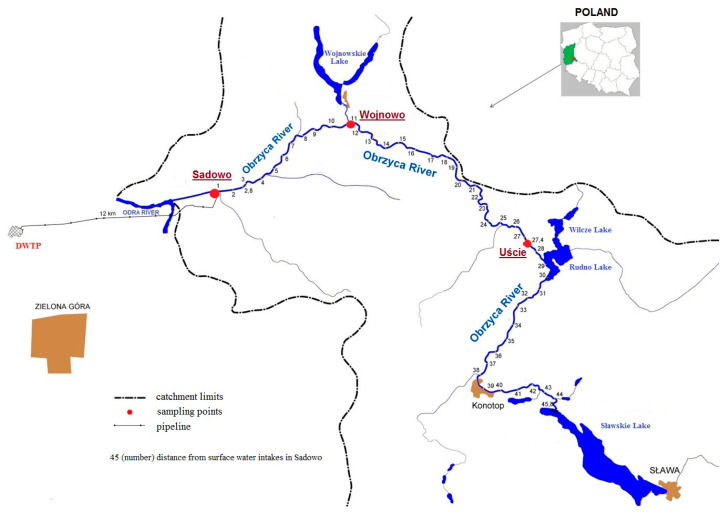
Test area.

The samples were taken from the Obrzyca River (central-western Poland) from July to October 2019. The Sławskie Lake is the beginning of the river, being the source of drinking water for inhabitants of the town of Zielona Góra (Lubuskie Province). The sample points were located at the following places of the river: Uście, Wojnowo, and Sadowo (Figure 3).

### 5.2. Sampling

The samples were collected once a month at three sampling points Sadowo, Uście, and Wojnowo (Figure 3) from July to October 2019, altogether 12 samples. The sample points were chosen as result of a previously conducted analysis of cyanotoxins in the river [9]. Samples for microscopic and toxicological analyses were taken using a plankton net with a mesh size of 10 µm [9,53]. For the other analyses, samples were collected in 1 L glass bottles.

### 5.3. Physical and Chemical Water Quality Indicator Analysis

The physical and chemical indicator analyses were carried out by laboratory assistants from the Water and Sewage Laboratory, WTP in Zielona Góra. The physical and chemical indicators of water quality and methods used in this research are presented in Table 5.

### 5.4. Microscopic Analysis

Algal enumeration was performed in triplicate using a Sedgewick–Rafter chamber and MN 358/A (OPTA TECH, Warszawa, Poland) microscope [9,53]. Biovolume results were presented as mm^3^/L.

### 5.5. Chlorophyll a

The spectrophotometric method was used for the determination of chlorophyll a. Briefly, 500 mL samples were filtered on GF/C filters and extracted with 90% acetone for 24 h and centrifuged for 10 min at 4000 rpm. The absorbance of supernatant was measured at 663 and 750 wavelengths, and 665 and 750 wavelengths after acidification with hydrochloric acid using a spectrophotometer DR 2000 (HACH, Germany). The concentration of the chlorophyll a was calculated according to the Polish Standardization Act [63].

### 5.6. Cyanotoxin Analysis

Water samples (250–500 mL) collected from the river were filtered through 47 mm fiberglass filter discs (Whatman GF/C), which had been stored at −20 °C prior to extraction and analyses. Cyanotoxin analyses were carried out at the University of Gdańsk (Analysis and Expertise Center) in the following way: methanol (90%) extracts from the material were prepared with a 15 min bath sonication (Sonorex, Bandelin, Berlin, Germany) followed by 1 min probe sonication with an HD 2070 Sonopuls ultrasonic disruptor equipped with an MS 72 probe (Bandelin, Berlin, Germany; 20 kHz, 25% duty cycle). After centrifugation at 10,000 g for 15 min, the supernatants were transferred to a chromatographic vial. The microcystins were analyzed using HPLC/DAD Agilent 1200 (Agilent Technologies Waldbronn, Germany) and LC-MS/MS systems (QTRAP5500, Applied Biosystems, Sciex, Concord, ON, Canada). The separation was carried out by a column chromatography Zorbax Eclipse XDB-C-18 (4.6 × 150 mm, 5 µm) (Agilent Technologies, Santa Clara, CA, USA). During the separation, a gradient elution mixture of two phases was used: 5% acetonitrile which contained 0.1% formic acid (A), and 100% acetonitrile containing 0.1% formic acid. The volume of the injection was 5 µL. Quantitative analysis was carried out using microcystins (MCs), anatoxin-a (ANA), cylindrospermopsin (CYN), and nodularin (NOD) standards from Alexis Biochemicals (Lausen, Switzerland) [26].

### 5.7. Bioassays Tests

Biotoxicological studies were carried out in the laboratory of the Department of Applied Ecology, University of Zielona Góra. Collected samples were measured volumetrically and centrifuged at 4000 rpm for 20 min. Precipitates were subjected to three freeze-and-thaw cycles to lyse the intact cells and release the intracellular toxins [64]. Cell lysis was confirmed microscopically. Before toxicological test volume was measured, the toxicity of 12 water samples was investigated. For all of them, toxicological tests using *Dugesia tigrina* were carried out. The obtained results were the basis for calculations of a lethal concentration, LC 50, expressed as % concentration of extracts. The method of the cultivation of the planarians for toxicological tests was developed [65]. *D. tigrina* reproduces sexually and asexually by nonsymmetric transverse fission. It also has an incredible ability to regenerate, which has been used for reproduction purposes of the species. Using the feature of planarians to regenerate, cultivation is carried out by artificial division (through cutting the body in two parts) [65].

**Figure 4:**
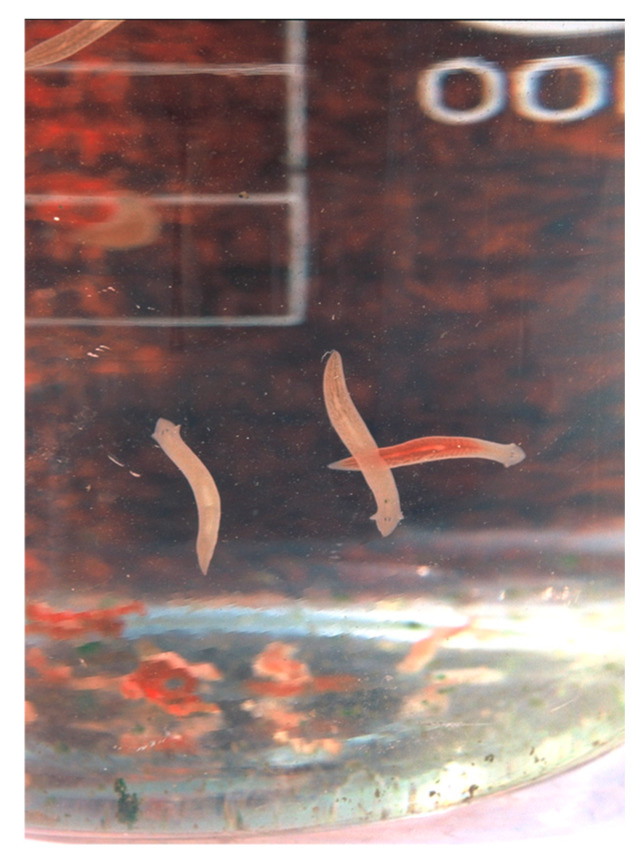
Cultures of planarians for biotoxicological studies [65].

The toxicological tests were performed in a laboratory at temperatures ranging between 20 and 22 °C. A series of 10 subsequent dilutions of the stock solution was prepared in a ratio to obtain concentrations of 100–4.8%. Test solutions with volumes of 40 mL were poured into beakers with capacities of 50 mL (Figure 4). Ten cut individuals were introduced into each beaker [28,65,66,67]. The determinations were carried out in three repetitions including control tests. Thirty planarians were kept in each concentration of the toxicant. After 240 h, the mortality of the planarians was checked. The obtained results constituted the basis for the calculation of a lethal concentration (240 h LC 50). The graphic interpretation method (probit analysis) was used to calculate the value of the concentrations of LC 50. The obtained results were subject to the test of compliance of experimental distribution with a normal distribution. The χ^2^ test was used in the calculations. The tested distributions were considered sufficiently convergent with the normal distribution if the likelihood the χ^2^ test was higher than 0.7 [68].

### 5.8. Statistical Analysis

Statistical analysis was performed using Excel 2010. The calculations included analysis of Pearson’s correlation and analysis of variance at the significance level α = 0.05. Chi-squared tests were used in order to check the probit graphical method.

## Figures and Tables

**Table 1:** Values of chlorophyll a concentration, cyanobacteria biovolume, and characteristics of dominant species including their percentage of its community in the Obrzyca River.

Sampling Months [month/year]	Chlorophyll a [µg/L]	Cyanobacteria Biovolume mm^3^/L]	Dominant * Species	Percentage of Cyanobacteria Biovolume
SADOWO				
07/2019	2.14	n.d.**	-	
08/2019	1.07	0.05	*Pseudanabaena limnetica*	100%
09/2019	3.20	0.12	*Pseudanabaena limnetica*	96%
10/2019	3.47	5.21	*Microcystis aeruginosa*	85%
UŚCIE				
07/2019	32.8	59.2	*Dolichospermum flos-aquae*	96%
08/2019	88.1	78.6	*Microcystis aeruginosa* *Dolichospermum flos-aquae*	49% 36%
09/2019	79.0	88.5	*Microcystis aeruginosa*	89%
10/2019	20.8	36.9	*Planktothrix agardhii*	69%
WOJNOWO				
07/2019	6.14	3.52	*Dolichospermum flos-aquae*	65%
08/2019	28.8	7.93	*Dolichospermum flos-aquae*	63%
09/2019	28.6	5.51	*Oscillatoria angusta*	76%
10/2019	7.21	1.06	*Oscillatoria angusta* *Microcystis wesenbergii*	47%31%

*Dominant species—constituting above 50% of the cyanobacteria community. The list of full species names of cyanobacteria cited in the manuscript is attached in Supplementary Materials; **n.d.—not detected.

**Table 2:** Cyanotoxin concentration in the Obrzyca River.

Sampling Months [month/year]	CYANOTOXINS [µg/L]										
	ANA	CYN	dmMC- RR	MC- RR	dmMC- LR	MC-LF	MC- LR	MC- LY	MC- LW	MC- YR	∑MCs
SADOWO											
07/2019	n.d.	n.d.	n.d.	n.d.	n.d.	n.d.	n.d.	n.d.	n.d.	n.d.	n.d.
08/2019	n.d.	n.d.	n.d.	n.d.	n.d.	n.d.	n.d.	n.d.	n.d.	n.d.	n.d.
09/2019	n.d.	n.d.	n.d.	<0.01	n.d.	n.d.	n.d.	n.d.	n.d.	n.d.	n.d.
10/2019	<0.01	n.d.	n.d.	0.01	0.73	0.03	0.06	<0.01	<0.01	n.d.	0.83
UŚCIE											
07/2019	n.d.	n.d.	n.d.	<0.01	n.d.	0.03	<0.01	n.d.	n.d.	n.d.	0.03
08/2019	n.d.	n.d.	n.d.	0.14	n.d.	0.06	0.32	<0.01	<0.01	0.09	0.61
09/2019	0.05	n.d.	1.56	0.58	n.d.	0.21	**1.33**	0.03	0.03	0.23	**3.97**
10/2019	0.02	n.d.	1.66	0.17	n.d.	0.06	0.23	0.01	0.01	0.07	2.21
WOJNOWO											
07/2019	0.01	n.d.	n.d.	<0.01	n.d.	0.07	<0.01	n.d.	n.d.	n.d.	0.07
08/2019	0.47	0.02	n.d.	<0.01	n.d.	0.01	n.d.	n.d.	<0.01	n.d.	0.01
09/2019	0.56	0.01	n.d.	0.3	0.41	0.02	n.d.	<0.01	<0.01	n.d.	0.46
10/2019	0.15	0.01	n.d.	0.01	0.73	n.d.	n.d.	<0.01	<0.01	0.13	0.86

n.d.—not detected, ANA—anatoxin-a, CYN—cylindrospermopsin, dm—demethylated forms of microcystins; ∑MCs—sum of microcystins. Maximal value of microcystins sum are bold and underlined. Concentrations above 1 μg/L are bold.

**Table 3:** Lethal concentration (LC 50) for *Dugesia tigrina* expressed as % concentration of analyzed extracts.

Sampling Months/Sites	SADOWO	UŚCIE	WOJNOWO
07/2019	n.t.	n.t.	n.t.
08/2019	n.t.	41.7%	n.t.
09/2019	n.t.	20.9%	n.t.
10/2019	n.t.	35.5%	n.t.

n.t.—not toxic.

**Table 4:** Values of Pearson correlation coefficients between cyanobacteria biovolume (Cya), cyanotoxins (MCs, ANA), and water quality indicators.

Water Quality Indicators	Cya	ANA	dmMC-RR	MC-RR	dmMC-LR	MC-LR	∑MCs
pH	0.69	0.00	−0.27	0.71	−0.16	0.70	0.55
NH_4_	0.36	0.17	0.31	−0.06	−0.37	−0.11	−0.05
Dissolved oxygen	0.24	0.38	−0.46	−0.24	0.20	−0.26	−0.31
P_tot_	0.43	−0.50	0.46	0.70	−0.40	0.70	0.68
N_tot_	0.60	−0.22	0.00	0.91	−0.43	0.91	0.77
PO_4_	0.07	−0.51	0.52	0.49	−0.33	0.49	0.51
Color	0.03	−0.31	0.41	0.33	−0.29	0.32	0.32
Turbidity	0.84	0.01	−0.23	0.79	−0.27	0.81	0.61
Total suspension	0.84	0.22	−0.09	0.77	−0.18	0.74	0.69
Water temperature	0.15	0.22	−0.36	−0.06	−0.46	−0.02	−0.39
N:P	0.02	0.47	−0.40	0.04	−0.05	0.03	−0.08

Statistically significant correlation coefficients (*p* < 0.05) are marked in bold, CYA—cyanobacterial abundance, ANA—anatoxin-a, MCs—microcystins.

**Table 5:** Physical and chemical parameters and methods used during the study.

Physical–Chemical Indicators	Measurement	Equipment	Standards
Ammonium nitrogen	Spectrophotometric	spectrometer DR 5000, Hach Germany	WAH HACH 2003 met. 8038 [54]
Color	Visual	-	EN ISO 7887:2011 [55]
Dissolved oxygen	Electrochemical	oxygen meter HQ30d, Hach Germany	EN ISO 5814:2012 [56]
Nitrate nitrogen	Ion chromatography	ion chromatograph 881 IC Compact Pro, Metrohm Switzerland	EN ISO 10304-1:2009 [57]
Orthophosphate	Spectrophotometric	spectrometer DR 5000, Hach Germany	WAH HACH 1997 met. 8048 [58]
pH	Electrochemical	pH meter 540 GLP, WTW Germany	EN ISO 10523:2012 [59]
Temperature	Electrochemical	oxygen meter HQ30d, Hach Germany	Manufacturer‘s instructions
Total nitrogen	Spectrophotometric	spectrometer DR 5000, Hach Germany	Cuvette Test LCK 138, Hach [60]
Total phosphorus	Spectrophotometric	spectrometer DR 5000, Hach Germany	Cuvette Test LCK 349, Hach [61]
Total suspended solids	Gravimetric	weight CP224S-OCE, Sartorius, Germany	EN 872:2005 [62]
Turbidity	Nephelometric	turbidimeter 2100 IS AN, Hach Germany	Manufacturer‘s instructions

## Supplementary Materials

The following are available online at https://www.mdpi.com/2072-6651/12/5/284/s1, Table S1. List of full species names cited in the manuscript. Table S2. Determination of 240-h LC 50 with *D. tigrina* for sample collected in August 2019 at Uście point, Table S1. Calculation of LC 50 by graphical method, Table S3. Checking the graphical method of calculating LC 50 using the *χ^2^* test, Table S4. Determination of 240-h LC 50 with *D. tigrina* for sample collected in September 2019 at Uście point, Table S2. Calculation of LC 50 by graphical method, Table S5. Checking the graphical method of calculating LC 50 using the *χ^2^* test, Table S6. Determination of 240-h LC 50 with *D. tigrina* for sample collected in October 2019 at Uście point, Table S3. Calculation of LC 50 by graphical method, Table S7. Checking the graphical method of calculating LC 50 using the *χ^2^* test.
