# Supplementary material for: The Occurrence of Potential Harmful Cyanobacteria and Cyanotoxins in the Obrzyca River (Poland), a Source of Drinking Water
Source: Toxins (Basel). 2020 Apr 28;12(5):284. doi: 10.3390/toxins12050284 (PMC7290984; doi:10.3390/toxins12050284)
Supplement: Supplementary file 1 [file toxins-12-00284-s001.pdf]

Supplementary Materia: The Occurrence of Potential Harmful Cyanobacteria and Cyanotoxins in the Obrzyca River (Poland), a Source of Drinking Water

Table S1. List of full species names cited in the manuscript.

Aphanizomenon flos-aquae (Ralfs ex Bornet & Flahault), *Cuspidothrix issatchenkoi* (Usachev)
Cylidrospermopsis raciborskii (Woloszynska)
Dolichospermum flos-aquae (Brébisson ex Bornet & Flahault)
Dolichospermum planctonicum (Brunnthaler)
Microcystis aeruginosa (Kützing)
Microcystis flos-aquae (Wittrock)
Microcystis viridis (A.Braun)
Microcystis wesenbergii (Komárek)
Oscillatoria angusta (Koppe)
Planktothrix agardhii (Anagn. & Komárek)
Pseudoanabaena limnetica (Lemmerman)
Woronichinia naegeliana (Unger)

Table S2. Determination of 240-h LC 50 with *D. tigrina* for sample collected in August 2019 at Uście point.

**Extract concentration [%]**	**Number of animals *n***	**Number of dead animals**		**Logarithm of concentration *x***	**Empirical probites *y***
		**Number of individuals *r***	**Percentage of individuals *P***		
**1.**	**2.**	**3.**	**4.**	**5.**	**6.**
71.4	30	21	70.00	1.85	5.524
51.02	30	18	60.00	1.71	5.2533
36.44	30	13	43.33	1.56	4.8318
26.03	30	10	33.33	1.42	4.5684
18.6	30	8	26.67	1.27	4.3781
13.28	30	5	16.67	1.12	4.0339
9.48	30	3	10.00	0.98	3.7184
6.7	30	1	3.33	0.83	3.1616

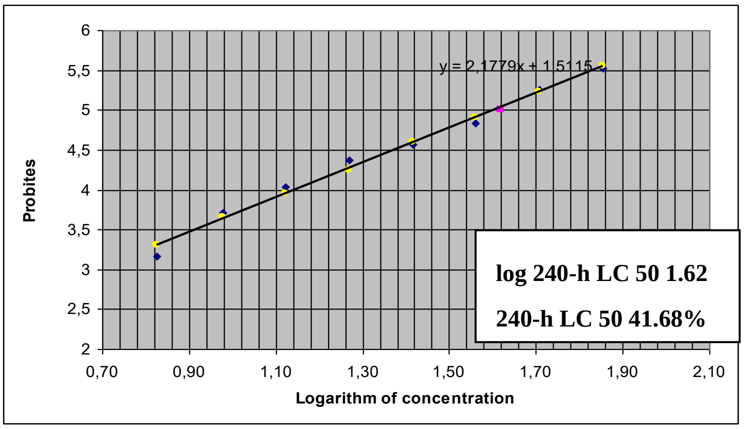

Figure S1. Calculation of LC 50 by graphical method.

Table S3. Checking the graphical method of calculating LC 50 using the χ^2 test.

Logarithm of concentration *X*	*y* theoretical	*P* theoretical	*n*	*r*	*nP*	*r-nP*	$\frac{(r-nP)^2}{nP(1-P)}$
**1.**	**2.**	**3.**	**4.**	**5.**	**6.**	**7.**	**8.**
1.85	5.55	0.709	30	21	21.27	−0.27	0.0118
1.71	5.23	0591	30	18	17.73	0.27	0.0101
1.56	4.9	0.461	30	13	13.83	−0.83	0.0924
1.42	4.60	0.345	30	10	10.35	−0.35	0.0181
1.27	4.25	0.227	30	8	6.81	1.19	0.2690
1.12	3.95	0.147	30	5	4.41	0.59	0.0925
0.98	3.65	0.089	30	3	2.67	0.33	0.0448
0.83	3.3	0.045	30	1	1.35	−0.35	0.0950
Number of degrees of freedom: f = 8−2 = 6. For χ^2 0,6337; *P* = 99%							0.6337

Table S4. Determination of 240-h LC 50 with *D. tigrina* for sample collected in September 2019 at Uście point.

Extract concentration [%]	Number of animals *n*	Number of dead animals: Number of individuals *r*	Number of dead animals: Percentage of individuals *P*	Logarithm of concentration *x*	Empirical probites *y*
**1.**	**2.**	**3.**	**4.**	**5.**	**6.**
71.4	30	28	93.33	1.85	6.499
51.02	30	26	86.67	1.71	6.1123
36.44	30	22	73.33	1.56	5.6219
26.03	30	16	53.33	1.42	5.0828
18.6	30	14	46.67	1.27	5.0828
13.28	30	7	23.33	1.12	4.9172
9.48	30	5	16.67	0.98	4.271
67	30	1	3.33	0.83	3.1616

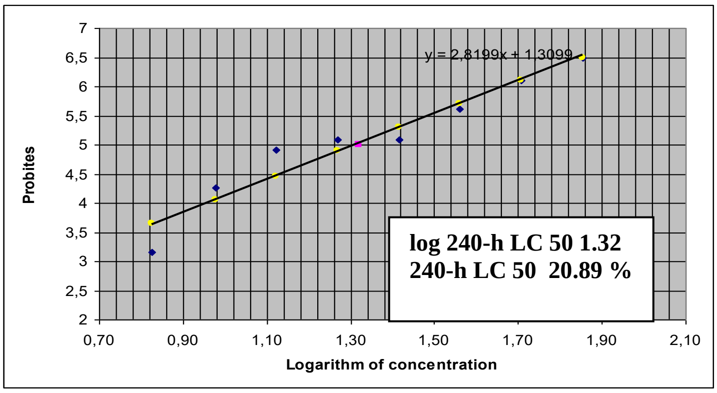

Figure S2. Calculation of LC 50 by graphical method.

Table S5. Checking the graphical method of calculating LC 50 using the χ^2 test.

Logarithm of concentration X	y theoretical	P theoretical	n	r	nP	r-nP	$\frac{(r-nP)^2}{nP(1-P)}$
**1.**	**2.**	**3.**	**4.**	**5.**	**6.**	**7.**	**8.**
1.85	6.5	0.934	30	28	28.02	−0.02	0.0002
1.71	6.1	0.865	30	26	25.95	0.05	0.0007
1.56	5.7	0.759	30	22	22.77	−0.77	0.1080
1.42	5.30	0.618	30	16	18.54	−2.54	0.9109
1.27	4.9	0.461	30	14	13.83	0.17	0.0039
1.12	4.45	0.292	30	7	8.76	−1.76	0.4994
0.98	4.05	0.172	30	5	5.16	−0.16	0.0060
0.83	3.65	0.089	30	1	2.67	−1.67	1.1466
Number of degrees of freedom: f = 8–2 = 6. For χ^2 2,6758; P = 80%							2.6758

Table S6. Determination of 240-h LC 50 with *D. tigrina* for sample collected in **October 2019** at Uście point.

Extract concentration [%]	Number of animals *n*	Number of dead animals: Number of individuals *r*	Number of dead animals: Percentage of individuals *P*	Logarithm of concentration *x*	Empirical probites *y*
**1.**	**2.**	**3.**	**4.**	**5.**	**6.**
71.4	30	25	83.33	1.85	5.966
51.02	30	18	60.00	1.71	5.2533
36.44	30	14	46.67	1.56	4.9172
26.03	30	10	33.33	1.42	4.5684
18.6	30	8	26.67	1.27	4.3781
13.28	30	5	16.67	1.12	4.0339
9.48	30	2	6.67	0.98	3.5015
6.7	30	1	3.33	0.83	3.1616

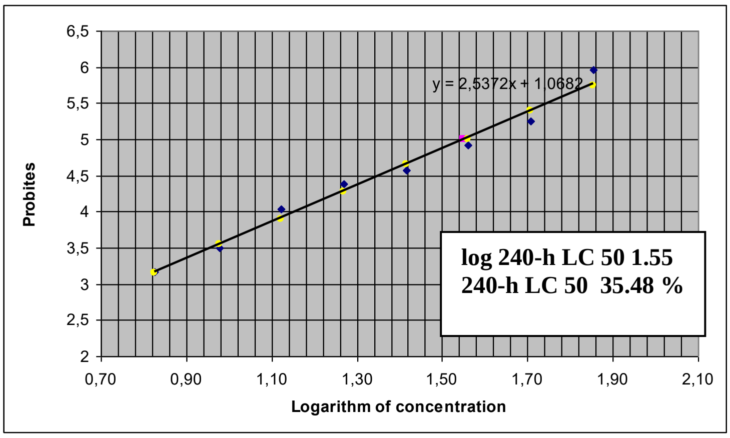

Figure S3. Calculation of LC 50 by graphical method.

Table S7. Checking the graphical method of calculating LC 50 using the χ^2 test.

**Logarithm of concentration** *X*	*y* **theoretical**	*P* **theoretical**	*n*	*r*	*nP*	*r-nP*	$\frac{(r-nP)^2}{nP(1-P)}$
**1.**	**2.**	**3.**	**4.**	**5.**	**6.**	**7.**	**8.**
1.85	5.75	0.774	30	25	23.22	1.78	0.6038
1.71	5.40	0.656	30	18	19.68	−1.68	0.4169
1.56	5.00	0.50	30	14	15	-1	0.1333
1.42	4.65	0.364	30	10	10.92	−0.92	0.1219
1.27	4.28	0.236	30	8	7.08	0.92	0.1565
1.12	3.90	0.136	30	5	4.08	0.92	0.2401
0.98	3.55	0.074	30	2	2.22	−0.22	0.0235
0.83	3.15	0.032	30	1	0.96	0.04	0.0017
Number of degrees of freedom: f = 8−2 = 6. For χ^2 1,6977; *P* = 94 %							1.6977
